# Observations on the Method of Curing the Hydrocele by Means of a Seton

**Published:** 1784

**Authors:** 


VI. *Obſervations on the method of curing the Hydrocele by means of a Seton. By* J. Howard, *Surgeon.* 8vo. Baldwin, *London*, 1783. 56 pages. 1 s. 6 d.

WIthin theſe few years the art of ſurgery has been enriched with two very uſeful methods of curing radically the hydrocele of the tunica vaginalis; namely, the ſeton and ſmall cauſtic. The advantages they evidently poſſeſs over inciſion, exciſion, and ſome other means which have been recommended and practiſed, are great and obvious; and are now ſo generally allowed, that they have been adopted by the generality of ſurgeons, as the ſafeſt and moſt certain methods of treatment. But even theſe, ſuperior as they undoubtedly are to thoſe which preceded them, might perhaps admit of ſome emendation, if the principles on which they operate in effecting a radical cure were thoroughly aſcertained and eſtabliſhed. The merits of theſe two methods are very fully and ably diſcuſſed in the tract now before us.

Mr.

Mr. Howard candidly acknowledges he has feen but few cafes treated by the fmall cauftic, having collected almoft all his ideas of it from the late Mr. Elfe's pamphlet on that fubject. He allows a great fhare of merit to this mode of treatment; but he cannot be brought to believe, that it will ever be fo extenfively ufeful as the feton. With regard to the latter he has been guided by long and repeated experience. Having for a confiderable number of years been employed, as an affiftant to Mr. Pott, in attending fome of his public, and a great number of his private patients, who have fubmitted to his method; he has had many opportunities of obferving the progrefs of the cures under different degrees of inflammation, and from thence has been induced to believe, that the inflammation raifed by the feton fhould be of fhort duration, and very moderate. He has, indeed, long been convinced, that the feton may be fo managed as to become itfelf, the regulator of that inflammation, which it is intended to produce; and that, merely by increafing or diminifhing the number of threads, it may be adapted to every poffible temperament, from the moft indolent to the moft irritable.

All the circumſtances under which a conſiderable diminution may be adviſeable he is not able to point out fully. But, he thinks, that whenever high inflammation is to be apprehended, eight or ten threads, or even a leſs number, may compoſe a ſeton of ſufficient thickneſs to anſwer the purpoſe of a radical cure, and that, even in old and full ſized hydroceles. The diameter of the conducting canula may be made, for theſe caſes, of the ſize of a common trocar canula uſed in the palliative method. When the tunica vaginalis contains but a ſmall quantity of fluid, more eſpecially if the habit is very irritable, he ſuppoſes, that a ſeton of the ſmalleſt ſize will probably be attended with the moſt favourable conſequences.

The uſual way of extracting the ſeton is by the inferior orifice. But having once obtained a radical cure within a fortnight, when he removed it from the ſuperior, he is inclined to think that the laſt is the beſt and moſt unexceptionable method. The lower orifice generally heals before the upper; and as the latter is not, from its ſituation, depending, a trifling lodgement of mucus will ſometimes keep it open for ſome days, and unavoidably protract the cure. The alteration alteration propofed will, our author believes, obviate this inconvenience.

